# Role of carotenoid β-cryptoxanthin in bone homeostasis

**DOI:** 10.1186/1423-0127-19-36

**Published:** 2012-04-02

**Authors:** Masayoshi Yamaguchi

**Affiliations:** 1 Department of Foods and Nutrition, The University of Georgia, 425 River Road, Rhodes Center, Room 448, Athens, GA 30602-2771, USA

**Keywords:** Carotenoid, β-cryptoxanthin, Osteoblastic bone formation, Osteoclastic bone resorption, Osteoporosis

## Abstract

Bone homeostasis is maintained through a balance between osteoblastic bone formation and osteoclastic bone resorption. Aging induces bone loss due to decreased osteoblastic bone formation and increased osteoclastic bone resorption. Osteoporosis with its accompanying decrease in bone mass is widely recognized as a major public health problem. Nutritional factors may play a role in the prevention of bone loss with aging. Among various carotenoids (carotene and xanthophylls including beta (β)-cryptoxanthin, lutein, lycopene, β-carotene, astaxanthin, and rutin), β-cryptoxanthin, which is abundant in Satsuma mandarin orange (*Citrus unshiu *MARC.), has been found to have a stimulatory effect on bone calcification *in vitro*. β-cryptoxanthin has stimulatory effects on osteoblastic bone formation and inhibitory effects on osteoclastic bone resorption *in vitro*, thereby increasing bone mass. β-cryptoxanthin has an effect on the gene expression of various proteins that are related osteoblastic bone formation and osteoclastic bone resororption *in vitro*. The intake of β-cryptoxanthin may have a preventive effect on bone loss in animal models for osteoporosis and in healthy human or postmenopausal women. Epidemiological studies suggest a potential role of β-cryptoxanthin as a sustainable nutritional approach to improving bone health of human subjects. β-Cryptoxanthin may be an osteogenic factor in preventing osteoporosis in human subjects.

## Introduction

Bone is a dynamic tissue that preserves skeletal size, shape, and structural integrity and to regulate mineral homeostasis. Bone homeostasis is maintained through a balance between osteoblastic bone formation and osteoclastic bone resorption. Aging and numerous pathological processes induce decrease in bone formation and increase in bone resorption, leading to osteoporosis, a devastating bone disease [1]. Osteoporosis, which is induced with decrease in bone mass, is widely recognized as a major public health problem [1]. The most dramatic expression of the disease is represented by fractures of the proximal femur for which the number increases as the population ages [2].

Nutritional factors may have the potential effect to prevent bone loss with increasing age. There is growing evidence that the supplementation of nutritional and food factors may have the preventive effect on bone loss that is induced in animal model of osteoporosis and in human subjects [3-6]. Chemical compounds in food and plants, which regulate on bone homeostasis, have been to be worthy of notice in maintaining of bone health and prevention of bone loss with increasing age [7-13].

Carotenoids (carotene and xanthophyll) are present in fruit and vegetables. Carotenoids, which are a provitamin A, may have an anabolic effect on bone metabolism. Vitamin A (retinol, retinal, and retinoic acid), which is formed from carotenoids in animal and human, has been shown to have a role in the regulation of bone cells and it may have an anabolic effect on bone [14-16]. However, vitamin A is also known to have a detrimental effect on bone at high doses [17-20]. In laboratory animals, high levels of vitamin A lead to accelerated bone resorption, bone fractures, and osteoporotic bone lesions [17].

Beta (β)-cryptoxanthin, a kind of xanthophyll, is abundant in Satsuma mandarin orange (*Citrus unshiu *MARC.). Of various carotenoids, β-cryptoxanthin has been found to have a potential-anabolic effect on bone due to stimulating osteoblastic bone formation and inhibiting osteoclastic bone resorption [21,22]. This review has been written to outline the recent advances concerning the role of β-cryptoxanthin in the regulation of bone homeostasis and in the prevention of osteoporosis, especially the cellular and molecular mechanisms by which β-cryptoxanthin stimulates osteoblastic bone formation and inhibits osteoclastic bone resorption.

### Effect of carotenoids in bone homeostasis

Bone is a dynamic tissue that undergoes continual adaptation during vertebrate life to attain and preserve skeletal size, shape, and structural integrity and to regulate mineral homeostasis. Bone homeostasis is regulated by the functions of osteoblasts, osteoclasts, and osteocytes which are major cells in bone tissues [23,24]. Osteoclasts, which develop from hematopoietic progenitors, are recruited to the site and excavate the calcified matrix. Osteoblasts arising from local mesenchymal stem cells assemble at the bottom of the cavity and bone formation begins. Bone acts as major storage site for growth factors [25]. Growth factors, which are produced by osteoblasts, diffuse into newly deposited osteoid and are stored in the bone matrix including isulin-like growth factors (IGF- I and II), transforming growth factor-β1 (TGF-β1), platelet-derived growth factor (PDGF), and bone morphologic protein (BMP). These bone-derived factors, which can be liberated during subsequent periods of bone resorption, act in an autocrine, paracrine, or delayed paracrine fashion in the local microenvironment of the bone surface. Thus, bone homeostasis is regulated through complex mechanism. Whether functional food factors can regulate bone homeostasis has been poorly understood.

Carotene, which is contained in plant and fruits, is generically named for carotene (α, β, γ, δ, ε) and lycopene, which is a natural compound with the derivative of fundamental form of C_40_H_56 _in chemical structure. Other derivatives with hydroxyl group for C_40_H_56 _are named as xanthophyll, which are lutein, zeaxanthin, canthaxanthin, fucoxanthin, antheraxanthin, violaxanthin, and β-cryptoxanthin. Carotenoids are named generically for carotene and xanthophyll.

The effects of carotenes, canthaxanthin, fucoxanthin, antheraxanthin, and violaxanthin on bone homeostasis have not been shown clearly. Among various carotenes and xanthophylls (including β-carotene, lycopene, lutein, astaxanthin, and β-cryptoxanthin), β-cryptoxanthin has been found to have a potential-anabolic effect on bone calcification *in vitro *[21,22]. Myricetin, kaempferol, isorhamnetin, curcumin, hesperidin, and rutin (quercetin-3-rutinoside) of various flavonoids do not have an effect on bone formation and calcification *in vitro *[22].

Vitamin A (retinol, retinal, and retinoic acid) is formed from carotenoids, which are provitamin A, in the animals and humans. The retinoic acid receptors (RAR) α, β and γ (RARα, RAR β and RARγ) are nuclear hormone receptors that regulate fundamental processes during embryogenesis, but their roles in skeletal development and growth are investigated.

Mice deficient in RARα and RARγ (or RAR β and RARγ) have been shown to exhibit severe growth retardation obvious by about 3 weeks postnatally [14]. Retinoic acid receptors may be required for skeletal growth, matrix homeostasis and growth plate function in postnatal mouse, suggesting a role of retinoic acid in bone growth [14].

Retinol and β-carotene have been shown to inhibit the proliferation of MC3T3-E1 cells as well as DNA synthesis of the cells in a dose-dependent manner [15] and stimulate differentiation of MC3T3-E1 cells by increasing alkaline phosphatase activity dose dependently [15]. α-Carotene, canthaxanthin, and lycopene also have been shown to inhibit MC3T3-E1 cell proliferation and to increase alkaline phosphatase activity and osteopontin mRNA expression [15].

The effects of retinoids on osteoclast differentiation in cultured mouse bone marrow cells (BMCs), bone marrow macrophages (BMMs), spleen cells, and RAW264.7 cells are shown by analyzing osteoclast formation and expression of important genes in signal transduction and osteoclast function [16]. All-*trans*-retinoic acid (ATRA) did not stimulate osteoclastogenesis in BMCs, but inhibited hormone and RANK (receptor activator of nuclear factor kappa B; NF-κB) ligand (RANKL)-induced gene expression and formation of osteoclasts [16]. ATRA abolished an increase in the transcription factors c-Fos and the nuclear factor of activated T cells (NFAT) NFAT2 stimulated by RANKL and suppressed down-regulation of the anti-osteoclastogenic transcription factor MafB [16].

Excessive intakes of vitamin A have been shown to have adverse skeletal effects in animals [17]. High vitamin A intake may lead to an increased risk of fracture in humans. Association between vitamin D deficiency and excess of vitamin A as a potential risk factor of osteoporosis and fracture is shown [18]. Whereas in women with vitamin D deficiency the risk of osteoporosis increased was up 5 times higher than women in the lowest quintile of retinol [18]. High retinol levels together with vitamin D deficiency may be hitherto an overlooked risk factor for osteoporosis.

Retinol is derived from both retinoids, which are contained in animal food, and carotenoids, which are contained in vegetables and fruits. A possible role of carotenoids in involutional osteoporosis is evaluated. When plasma levels of β-carotene and other carotenoids, in addition to those of retinol, were measured in free-living, non-supplemented, elderly women with or without severe osteoporosis, plasma levels of retinol and of all carotenoids tested, with the exception of lutein, were consistently lower in osteoporotic than in control women [26]. A weak association was found only between retinol and femoral neck bone mineral density in osteoporotic women [26]. A bone sparing effect of retinol, to which the provitamin A activity of some carotenoids, may be contributed [26].

Carotenoid lycopene has been shown to have an inhibitory effect on bone resorption *in vitro *[27], although lycopene is not shown to have a stimulatory effect on mineralization using rat femoral tissues *in vitro *[21]. Lycopene (10^-5 ^M) has been found to inhibit basal and parathyroid hormone (PTH)-stimulated osteoclastic mineral resorption and formation of tartrate-resistant acid phosphatase (TRAP) activity-possitive multinucleated osteoclasts [27]. Lycopene may have an inhibitory effect on osteoclastic bone resorption, suggesting that lycopene has a role in the prevention of osteoporosis.

In a cross-sectional study by using 33 postmenopausal women aged 50-60 years, groups with higher lycopene intake, as determined from the dietary records, showed higher serum lycopene [28]. A higher serum lycopene has been found to be associated with a low cross-linked *N*-telopeptides of type I collagen [28]. Similarly, groups with higher serum lycopene have lower protein oxidation [28]. These observations suggest that the dietary antioxidant lycopene reduces oxidative stress and the levels of bone turnover markers in postmenopausal women, and may be beneficial in reducing the risk of osteoporosis [28].

Postmenopausal women supplemented with lycopene are shown to increase antioxidant capacity and decrease oxidative stress and the bone resorption marker *N*-telopeptide [29]. Lycopene decreases bone resorption markers and may reduce the risk of osteoporosis [29]. Sixty postmenopausal women, 50-60 years old, were recruited [29]. Following a 1-month washout without lycopene consumption, participants consumed either regular tomato juice, lycopene-rich tomato juice, tomato lycopene capsules, or placebo capsules, twice daily for total lycopene intakes of 30 and 70 mg/day respectively for 4 months [29]. Lycopene supplementation for 4 months significantly increased serum lycopene and decreased lipid peroxidation, and cross-linked aminoterminal *N*-telopeptide as compared to placebo [29]. The antioxidant lycopene may be beneficial in reducing oxidative stress parameters and the bone resorption marker [29].

### β-Cryptoxanthine and bone homeostasis

Xanthophyll β-cryptoxanthin, which is a kind of carotenoid, is abundant in Satsuma mandarin orange (*Citrus unshiu *MARC). The molecular weight of β-cryptoxanthin is 552. The biological function of β-cryptoxanthin in animal and human, however, has been not determined fully. Yamaguchi et al. found that β-cryptoxanthin has been shown to have a unique anabolic effect on bone calcification; such an effect is not seen in lutein, lycopene, or astaxanthin, which is other carotenoids and flavonoid rutin (quercetin-3-rutinoside) [21,22].

#### β-Cryptoxanthin stimulates bone formation and inhibits bone resorption in bone tissue culture

β-Cryptoxanthin has been shown to have a unique anabolic effect on bone calcification. Culture with β-cryptoxanthin (10^-7 ^or 10^-6 ^M) has been found to cause an increase in calcium content and alkaline phosphatase activity in the femoral-diaphyseal (cortical bone) and -metaphyseal (trabecular bone) tissues *in vitro*. Lutein, lycopene, and rutin (10^-8 ^to 10^-6 ^M) did not have anabolic effects on alkaline phosphatase activity and calcium contents in rat femoral tissues [21]. Astaxanthin and β-carotene did not have an effect on the femoral calcium contents [22]. Myricetin, kaempferol, isorhamnetin, curcumin, or hesperidin (10^-7 ^to 10^-5 ^M) had no effect on bone calcium content in tissue cultures *in vitro *[22]. Quercetin significantly increased calcium content in femoral diaphyseal tissues but not metaphyseal tissues. β-Cryptoxanthin has a unique anabolic effect on bone calcification *in vitro*. The effect of β-cryptoxanthin increasing bone components was completely prevented in the presence of cycloheximide, an inhibitor of protein synthesis, suggesting that the effect is needed newly protein synthesis [22].

β-Cryptoxanthin has also been shown to inhibit bone resorption in bone tissue cultures *in vitro *[22]. Parathyroid hormone (PTH) or prostaglandin E_2 _(PGE_2_), which is a bone-resorbing factor, can stimulate osteoclastic bone resorption *in vitro *[30-32]. Culture with PTH or PGE_2 _caused a decrease in calcium content in the diaphyseal and metaphyseal tissues [22]. This decrease was completely inhibited in the presence of β-cryptoxanthin (10^-8 ^to 10^-6 ^M). Likewise, culture with β-cryptoxanthin completely inhibited the PTH- or PGE_2_-induced increase in medium glucose consumption and lactic acid production by bone tissues [22]. β-Cryptoxanthin has an inhibitory effect on bone resorption in tissue culture *in vitro*.

Thus, β-cryptoxanthin has been shown to have stimulatory effect on osteoblastic bone formation and inhibitory effects on osteoclastic bone resorption in bone tissue culture *in vitro*. Serum concentration of β-cryptoxanthin due to consumption of vegetable juice in women is shown to be in the range of 1.3 × 10^-7 ^to 5.3 × 10^-7 ^M [33]. β-Cryptoxanthin in the range of 10^-8 ^to 10^-6 ^M has an anabolic effect on biochemical components in rat femoral tissues *in vitro*, suggesting a physiologic role in the regulation of bone metabolism.

#### β-Cryptoxanthin stimulates osteoblastogenesis

The cellular and molecular mechanisms by which β-cryptoxanthin stimulates bone formation in bone tissues has been examined by using osteoblastic MC3T3-E1 cells *in vitro*. β-Cryptoxanthin has been found to stimulate the proliferation of osteoblastic cells in subconfluent monolayers in a medium containing 10% fetal bovine serum [34]. Culture with β-cryptoxanthin also caused an increase in biochemical components (alkaline phosphatase activity, protein, and DNA contents) of osteoblastic cells [34]. This effect was abolished in the presence of staurosporine, an inhibitor of protein kinase C, or PD98059, an inhibitor of mitogen- activated protein kinase (MAPK), although the effect of β-cryptoxanthin in increasing cellular biochemical components was not prevented in culture with dibucain, an inhibitor of Ca^2+^/calmodulin-dependent protein kinase [34]. The stimulatory effect of β-cryptoxanthin on osteoblastic cell components seems to be partly mediated through signaling factors of protein kinase C or MAPK in the cells.

The effects of β-cryptoxanthin in increasing the biochemical components in osteoblastic cells were completely inhibited in the presence of 5,6-dichloro-1- β-D-ribofuranosylbenzimidazole (DRB), an inhibitor of RNA polymerase II, suggesting that the effect of carotenoid results from a stimulatory effect on transcriptional activity in osteoblastic cells.

The mineralization in osteoblastic cells is shown to stimulate in the prolonged culture with β-cryptoxanthin [35]. The stimulatory effect of β-cryptoxanthin on mineralization may result from the carotenoid-induced proliferation and differentiation of osteoblastic cells.

β-Cryptoxanthin may stimulate gene expression for proteins that are involved in bone formation and mineralization in osteoblastic cells. The effect of β-cryptoxanthin on gene expression in osteoblastic cells using reverse transcription-polymerase chain reaction (RT-PCR) is examined. Culture with β-cryptoxanthin was found to stimulate the mRNA expression of IGF-I or TGF- β1 in osteoblastic cells [34]. This finding may support the view that β-cryptoxanthin has a stimulatory effect on transcriptional activity in osteoblastic cells. IGF-I or TGF-β1 is a bone growth factor produced from osteoblasts [36-38]. The stimulatory effect of β-cryptoxanthin on the proliferation of osteoblastic cells may be partly mediated through the action of IGF-I or TGF-β1 produced from the cells.

TGF-β1 has a potent activity on osteoblast-lineage commitment an event that is partly mediated through Smad transcription factors [38]. It has been reported that NF-κB signaling represses basal osteoblast differentiation and mineralization in MC3T3 cells and antagonizes TGF-β1 and BMP-2 mediated MC3T3 mineralization by downregulating Smad activation [39]. Interestingly, β-cryptoxanthin has been shown to have the capacity to suppress the receptor activator of nuclear factor-kappa B (NF-κB) activity in MC3T3 preosteoblastic cells [40], suggesting that one mechanism by which β-cryptoxanthin may stimulate osteoblast differentiation may be by promoting Smad activation. This finding also suggests that β-cryptoxanthin may lead to enhanced Smad signaling. While, β-cryptoxanthin failed to directly stimulate Smad activity, it did however amplify TGF-β1-induced Smad signaling [41].

Furthermore, whether β-cryptoxanthin directly stimulates Smad activation or regulates TGF-β1-induced Smad activation in osteoblastic cells *in vitro *is examined [41]. β-Cryptoxanthin was found to potentiate TGF-β1-induced, but not BMP-2-induced, Smad activation in MC3T3 preosteoblastic cells, suggesting that one mechanism by which β-cryptoxanthin stimulates bone formation is by potentiating the TGF-β1-mediated commitment of preosteoblasts to differentiate along the osteoblastic pathway [41].

Runx2 (Cbfa1) is a member of the runt domain family of transcription factors and a master regulator of osteoblast differentiation [42]. α1 (I) Collagen is a matrix protein that is related to bone formation and mineralization in osteoblast lineage cells [43]. Alkaline phosphatase participates in the mineralization process in osteoblastic cells [44]. β-Cryptoxanthin (10^-7 ^or 10^-6 ^M) was found to increase the mRNA expression of Runx2, α1 (I) collagen, and alkaline phosphatase in osteoblastic MC3T3-E1 cells [35].β-Cryptoxanthin had a stimulatory effect on the gene expression of various proteins involved in osteoblasic bone formation [35]. Such effects of β-cryptoxanthin were blocked in the presence of DRB [34], supporting the view that the carotenoid stimulates transcriptional activity in osteoblastic MC3T3-E1 cells.

Vitamin A (retinol) may be able to bind to nuclear receptors in cells. Retinol and β-carotene has been shown to inhibit the proliferation of osteoblastic MC3T3-E1 cells as well as DNA synthesis of the cells, due to increasing alkaline phosphatase activity dose dependently (10^-9 ^to 10^-7 ^M) [15]. Vitamin A (10^-7 ^or 10^-6 ^M) increases alkaline phosphatase activity in osteoblastic cells [34]. β-Cryptoxanthin (10^-7 ^or 10^-6 ^M) caused an increase in alkaline phosphatase activity and protein content in osteoblastic cells. This effect was also seen in the presence of vitamin A (10^-6 ^M) [34]. Moreover, the stimulatory effect of β-cryptoxanthin on the expression of Runx2 type 1 and α1 (I) collagen mRNA was observed in the presence of vitamin A [34]. Vitamin A did not have a significant effect on Runx2 type 1 mRNA expression in osteoblastic MC3T3-E1 cells [34]. Thus, the mode of action of β-cryptoxanthin on gene expression in osteoblastic cells may differ from that of vitamin A, which is mediated through the retinoid X receptor (RXR) in the nucleus of the cells [34].

**Figure 1:**
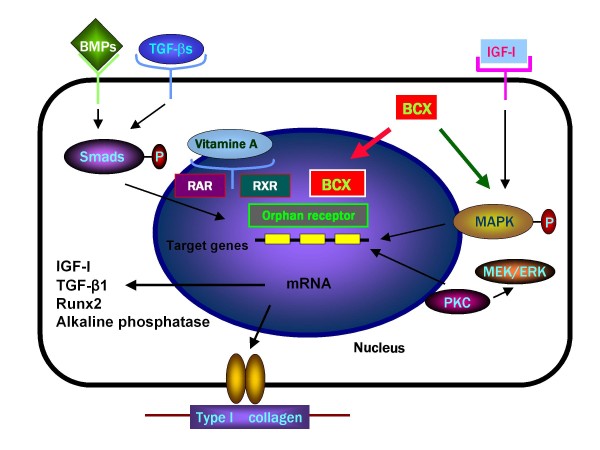
**The cellular and molecular mechanism by which β-cryptoxanthin (BCX) stimulates bone formation and mineralization in osteoblastic cells**. BCX may bind to orphan receptors in the nucleus of osteoblastic cells, and it stimulates gene expression of bone formation-related proteins. BCX also stimulates nuclear transcriptional activity mediated through activation of protein kinase C (PKC) or mitogen-activated protein kinase (MAPK) in osteoblastic cells.

It is speculated that β-cryptoxanthin can bind to other receptors (including orphan receptors that ligands are not unknown), and that the carotenoid may stimulate transcriptional activity in osteoblastic cells. The mechanism of β-cryptoxanthin action in stimulating proliferation, differentiation, and mineralization in osteoblastic cells is summarized in Figure 1.

#### β-Cryptoxanthin suppresses osteoclastogenesis

NF-κB ligand (RANKL) plays a pivotal role in osteoclastogenesis from bone marrow cells. RANKL expression is induced in osteoblastic cells and bone marrow stromal cells in response to osteoporotic factors, such as PTH, PGE_2_, and 1,25-dihydroxyvitamin D_3 _(VD_3_), and combined treatment of hematopoietic cells with macrophage colony-stimulating factor (M-CSF) and the soluble form of RANKL (sRANKL) induce osteoclast differentiation in vitro [45,46]. The receptor protein RANK (receptor activator of NF-κB) is expressed on the surface of osteoclast progenitors.

β-Cryptoxanthin (10^-8 ^to 10^-6 ^M) is shown to have a potent inhibitory effect on osteoclast-like cell formation in mouse marrow culture *in vitro *[47]. The inhibitory effect of *β*-cryptoxanthin on osteoclast-like cell formation was seen at the later stage of osteoclast differentiation in bone marrow cultures [47]. Culture with β-cryptoxanthin caused a marked inhibition of osteoblast-like cell formation induced in the presence of PTH, PGE_2_, VD_3_, lipopolysaccharide, or tumor necrosis factor-α (TNF-α). β-Cryptoxanthin also had an inhibitory effect on osteoclast-like cell formation induced by RANKL [47]. The inhibitory effect of β-cryptoxanthin was equal to that of 17 β-estradiol (E_2_), calcitonin, genistein, and zinc sulfate, which can inhibit osteoclast-like cell formation induced by bone-resorbing factors [47].

The interaction of RANKL with its receptor RANK leads to the recruitment of the signaling adaptor molecules TRAFs (TNF receptor-associated factors) to the receptor complex and the activation of NF-κB and c-Jun N-terminal kinase (JNK) [48,49]. Protein kinase C family enzyme has a role in regulation of osteoclast formation and function potentially by participating in the extracellular signaling-regulated kinase (ERK) signaling pathway of M-CSF and RANKL [49].

Phorbol 12-myristate 13-acetate (PMA), an activator of protein kinase C, stimulated osteoclast-like cell formation in mouse marrow cultures, and the PMA-induced osteoclastogenesis was found to inhibit in the presence of β-cryptoxanthin [47]. Moreover, β-cryptoxanthin had an inhibitory effect on dibutyryl cyclic adenosine monophosphate (DcAMP)-induced osteoclast-like cell formation in mouse marrow cultures [47]. It is assumed that activation of protein kinase C and protein kinase A pathways leads to increased RANKL expression, and that β-cryptoxanthin can inhibit protein kinase C- or protein kinase A-related RANKL expression in osteoclastogenesis.

The effect of β-cryptoxanthin on mature osteoclasts is demonstrated [50]. M-CSF-dependent bone marrow macrophages were cultured in the presence of M-CSF and RANKL for 4 days [50]. The osteoclastic cells formed were further cultured in medium containing β-cryptoxanthin with or without M-CSF and RANKL for 24-72 hours. The number of osteoclastic cells has been found to decrease in culture with β-cryptoxanthin (10^-7 ^or 10^-6 ^M) in the presence or absence of M-CSF and RANKL for 72 hours. The β-cryptoxanthin-induced decrease in osteoclastic cells was inhibited in the presence of caspase-3 inhibitor. The results of agarose gel electrophoresis showed the presence of low-molecular-weight DNA fragments of adherent cells cultured with β-cryptoxanthin. These findings indicate that the carotenoid induces apoptotic cell death.

Apoptosis-related gene expression was determined by using RT-PCR [50]. The expression of caspase-3 mRNA or Apaf-2, which involves apoptosis, in osteoclastic cells was found to stimulate when cultured with β-cryptoxanthin in the presence or absence of M-CSF and RANKL [50]. β-Cryptoxanthin-induced apoptotic cell death may be partly mediated through caspase-3 expression in osteoclastic cells.

The expression of Bcl-2 mRNA, which is involved in the rescue of apoptosis, was decreased after culture with β-cryptoxanthin in the presence or absence of M-CSF and RANKL [50]. However, Akt-1 mRNA expression was not significantly changed in culture with β-cryptoxanthin. The decrease in Bcl-2 mRNA expression may partly contribute to the effect of β-cryptoxanthin in stimulating the apoptotic cell death of osteoclastic cells.

Culture with β-cryptoxanthin is found to have suppressive effects on tartrate-resistant acid phosphatase (TRACP) activity, and it decreases TRACP and cathepsin K mRNA expressions in osteoclastic cells in the presence or absence of M-CSF and RANKL [50]. These findings suggest that β-cryptoxanthin can inhibit the enhancement of bone-resorbing activity in osteoclasts. β-Cryptoxanthin inhibitd various bone-resorbing factors-induced decrease in bone calcium content and increase in lactic acid production in rat femoral tisuue culture system *in vitro*. Presumably, β-cryptoxanthin has an inhibitory effect on the activation of mature osteoclasts.

**Figure 2:**
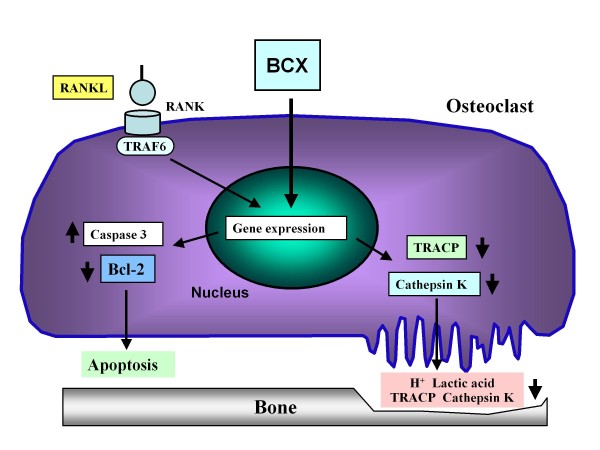
**The molecular mechanism by which β-cryptoxanthin (BCX) has suppressive effects on mature osteoclasts**. BCX inhibits osteoclast formation from mononuclear osteoclasts which are mediated through RANKL and RANK signaling in bone marrow culture systems. BCX stimulates apoptotic cell death by stimulating the gene expression of caspase-3, an apoptosis-inducing enzyme, and suppression of gene expression of Bcl-2, a rescue protein of apoptosis. In addition, BCX suppresses the gene expression of TRACP and cathepsin K, which are bone resorption-related enzymes, and their enzyme activities in mature osteoclasts.

As mentioned above,β-cryptoxanthin has stimulatory effects on apoptotic cell death due to activating gene expression of its related proteins. The carotenoid also has suppressive effects on TRACP activity and gene expression of enzymes that involve in bone-resorbing activity in osteoclastic cells. The action of β-cryptoxanthin in osteoclasts is summarized in Figure 2.

### Effect of β-cryptoxanthin on bone is synergistically enhanced by zinc

Zinc, an essential trace element, has an anabolic effect due to stimulating osteoblastic bone formation and osteoclastic bone resorption in vitro and in vivo [6,51-55]. The effect of the combination of β-cryptoxanthin and zinc sulfate on bone components in the femoral-diaphyseal and -metaphyseal tissues of young rats in vitro is examined [56]. Bone tissues were cultured for 48 hours in a serum-free medium containing either vegicle, β-cryptoxanthin (10^-9^-10^-7 ^M) or zinc sulfate (10^-6^-10^-4 ^M). The presence of β-cryptoxanthin (10^-9 ^M) or zinc (10^-6 ^M) did not have a significant effect on calcium content in the femoral-diaphyseal or -metaphyseal tissue [56]. However, culture with combination of β-cryptoxanthin (10^-9 ^M) and zinc (10^-6 ^M) caused an increase in calcium content in the femoral-diaphyseal and -metaphyseal tissues. Such an effect was not observed in the combination of β-cryptoxanthin (10^-9 ^M) plus genistein (10^-6 ^M) or menaquinone-7 (10^-6 ^M), or zinc (10^-6 ^M) plus genistein (10^-6 ^M) or menaquinone-7 (10^-6 ^M) [56].

The combination of β-cryptoxanthin (10^-9 ^M) plus zinc (10^-6 ^M) was found to cause a remarkable increase in alkaline phosphatase activity and DNA content in the diaphyseal and metaphyseal tissues, while their application alone did not have an effect on the enzyme activity or DNA content in the bone tissues [56]. The effect of the combination of β-cryptoxanthin (10^-9 ^M) plus zinc (10^-6 ^M) in increasing alkaline phosphatase activity, DNA and calcium contents in the diaphyseal and metaphyseal tissues was completely prevented in the presence of cycloheximide, an inhibitor of protein synthesis, or DBR, an inhibitor of transcriptional activity [56].

#### Synergistic effect of β-cryptoxanthin and zinc in osteoblastogenesis

The effect of the combination with β-cryptoxanthin and zinc or other factors [including 1,25-dihydroxyvitamin D_3 _(VD_3_), 17 β-estradiol, genistein, or menaquinone-7 (MK-7)] on gene expression in osteoblastic MC3T3-E1 cells is examined [57]. Culture with β-cryptoxanthin (10^-7 ^or 10^-6 ^M) caused an increase in the expression of Runx2 and alkaline phosphatase mRNAs in the cells. This effect was not enhanced in the presence of VD_3_, 17 β-estradiol, genistein, or MK-7. Culture with zinc sulfate (10^-5 ^M) caused an increase in α 1 (I) collagen mRNA expression, while it did not have a significant effect on Runx2 or osteocalcin mRNA expression in the cells. The effect of β-cryptoxanthin (10^-7 ^M) in increasing Runx2 or α 1 (I) collagen mRNA expressions was enhanced in the presence of zinc (10^-6 ^or 10^-5 ^M) [57]. Such an effect was not seen in the presence of cycloheximide or DRB. The additive and/or synergistic effect of β-cryptoxanthin and zinc on gene expression in osteoblastic cells is partly resulted in newly synthesized protein components.

Zinc activates aminoacyl-tRNA synthetase, a rate-limiting enzyme, in the translational process of protein synthesis [53-55]. β-Cryptoxanthin can stimulate transcriptional activity in osteoblastic cells [34]. It is speculated that β-cryptoxanthin stimulates gene expression and zinc increases translational activity in osteoblastic cells. This may be important as a possible mechanism by which zinc enhances the anabolic effect of β-cryptoxanthin in osteoblastic cells.

#### Synergistic effect of β-cryptoxanthin and zinc in osteoclastic cells

The effect of β-cryptoxanthin on osteoclastic cells formed in the mouse marrow culture system in vitro was found to enhance after culture with zinc [58]. Bone marrow cells were isolated from mice. The macrophage colony-stimulating factor (M-CSF)-dependent bone marrow cells were cultured in the presence of M-CSF and RANKL for 96 hours. The osteoclastic cells formed were further cultured for 24 or 72 hours in a medium containing either vehicle, β-cryptoxanthin, zinc sulfate, or β-cryptoxanthin plus zinc with or without M-CSF and RANKL. The number of osteoclastic cells was decreased after culture with the combination of β-cryptoxanthin (10^-7 ^M) and zinc (10^-5 ^M) in the presence or absence of M-CSF and RANKL for 24 or 72 h as compared with the value for β-cryptoxanthin or zinc [58].

The results of agarose gel electrophoresis showed the presence of low-molecular weight deoxyribonucleic acid (DNA) fragments of adherent cells cultured with β-cryptoxanthin plus zinc for 24 or 72 hours in the presence of M-CSF and RANKL, indicating that the combination of the two chemicals synergistically induces apoptotic cell death [58].

β-Cryptoxanthin plus zinc-induced decrease in osteoclastic cells was inhibited in the presence of caspase-3 inhibitor [58]. Culture with β-cryptoxanthin plus zinc for 24 or 72 hours caused an increase in caspase-3 mRNA expression in the presence or absence of M-CSF and RANKL as compared with the value for each chemical alone. β-Cryptoxanthin plus zinc-induced increase in caspase-3 mRNA expression was completely inhibited in the presence of cycloheximide or DRB [58]. This suggests that β-cryptoxanthin plus zinc-induced apoptotic cell death is mediated through caspase-3 in osteoclastic cells, and that β-cryptoxanthin plus zinc-enhanced caspase-3 mRNA expression in osteoclastic cells is related to newly synthesized protein synthesis.

The mRNA expression of tartrate-resistant acid phosphatase (TRACP) and cathepsin K was decreased after culture with β-cryptoxanthin plus zinc in the presence or absence of M-CSF and RANKL for 72 hours as compared with β-cryptoxanthin or zinc alone [58].

Nuclear factor of activated T cells c1 (NFATc1) mRNA expression was decreased after culture with β-cryptoxanthin plus zinc in the presence or absence of M-CSF and RANKL for 72 hours as compared with each chemical alone, while NF-κB mRNA expression was not changed [58]. TRACP and cathepsin K are enzymes that are involved in the degradation of bone matrix components, and their enzyme activities are increased in RANKL-stimulated bone resorption [45,46]. The combination of β-cryptoxanthin and zinc may have a potent-suppressive effect on bone resorption.

NF-κB and NFATc1 are molecules related to RANKL signaling [45,46]. NFATc1 is a transcriptional factor that enhances the gene expression of TRACP and cathepsin K in osteoclasts, and the binding of NFATc1 to promoter is involved in NF-κB or AP-1 [45]. The suppression of NFATc1 mRNA expression induced with the combination of β-cryptoxanthin and zinc may induce the decrease in NF-κB protein level. This may partly contribute to the decrease in the TRACP or cathepsin K mRNA expression caused by their combination.

Thus, the suppressive effects of β-cryptoxanthin on osteoclastogenesis was demonstrated to enhance synergistically in the presence of zinc, and also the combination of β-cryptoxanthin and zinc has potent suppressive effects on osteoclastic cell function *in vitro*.

#### Anabolic effect of β-cryptoxanthin on bone is enhanced by zinc in vivo

The effects of combined β-cryptoxanthin and zinc on bone components in the femoral-diaphyseal (cortical bone) and -metaphyseal (trabecular bone) tissues of rats in vivo is shown [59]. Rats were orally administered either vehicle, β-cryptoxanthin (50 or 100 μg/kg body weight), zinc sulfate (1 or 5 mg Zn/kg), or their combination once a day for 7 days. Alkaline phosphatase activity, DNA and calcium contents in the femaral-diaphyseal tissues were not altered after the administration of β-cryptoxanthin (50 μg/kg) or zinc (1 or 5 mg/kg) [59]. Combined administration of β-cryptoxanthin (50 μg/kg) and zinc (1 or 5 mg/kg) caused a synergistic increase in alkaline phosphatase activity, DNA and calcium contents in the diaphyseal tissues [59]. The effect of β-cryptoxanthin (50 or 100 μg/kg) in increasing DNA and calcium contents in the metaphyseal tissues was enhanced after the combined administration of zinc (1 or 5 mg/kg), but it did not have an effect on the metaphyseal components. The metaphyseal alkaline phosphatase activity has been found to increase markedly after the administration of the combination of β-cryptoxanthin (50 μg/kg) and zinc (1 or 5 mg/kg) [59]. Study demonstrates that the oral administration of the combination of zinc at lower doses synergistically enhances β-cryptoxanthin-induced anabolic effects on the femoral tissues of rats in vivo. It is speculated that the combination of β-cryptoxanthin and zinc has stimulatory effects on the gene expression, protein synthesis, and cell proliferation in osteoblastic cells in rat femoral tissues. This may contribute to their enhancing effect on bone components in femoral tissues of rats in vivo.

The combination of β-cryptoxanthin plus zinc at a lower concentration has a synergistic effect on bone components *in vivo*. The combination of β-cryptoxanthin plus zinc has potentiality in the prevention of bone loss with aging. This finding is interested in respect of the development of new supplement with the composition of food factors that reveal a potent-anabolic effect in prevention of osteoporosis. It also would be useful to identify some of the foods that contain higher levels of β-cryptoxanthin and zinc.

### Preventive effect of β-cryptoxanthin on bone loss in vivo

As mentioned above, β-cryptoxanthin has been shown to have a stimulatory effect on osteoblastic bone formation and an inhibitory effect on osteoclastic bone resorption *in vitro*. Furthermore, the preventive effect of β-cryptoxanthin on osteoporosis is demonstrated by using animal models *in vivo*.

The anabolic effect of β-cryptoxanthin on bone components in young and aged rats is examined. β-Cryptoxanthin (100, 250, or 500 μg/kg body weight) was orally administered once daily for 7 days to young male rats [60]. The administration of β-cryptoxanthin (250, or 500 μg/kg) caused an increase in alkaline phosphatase activity, DNA and calcium contents in the femoral-diaphyseal and -metaphyseal tissues [61]. Such an effect was also observed in the femoral tissues of aged (50-week-old) female rats [62]. β-Cryptoxanthin has been shown to have an anabolic effect on bone components in rats in vivo.

β-Cryptoxanthin has been sown to have a preventive effect on bone loss in the pathophysiologic state. Bone loss is induced in streoptozotocin (STZ)-diabetic rats [62]. Young rats received a single subcutaneous administration of STZ (60 mg/kg body weight), and then the animals were orally administered β-cryptoxanthin (50 or 100 μg/kg) once daily for 7 or 14 days. The administration of STZ caused a decrease in body weight and a significant increase in serum glucose, triglyceride, and calcium levels, indicating a diabetic state [62]. These alterations were prevented after the administration of β-cryptoxanthin (50 or 100 μg/kg) for 14 days [62]. Alkaline phosphatase activity, DNA and calcium contents in the femoral-diaphyseal and -metaphyseal tissues were decreased in STZ-diabetic rats [62]. These decreases were prevented after the administration of β-cryptoxanthin (50 or 100 μg/kg) for 14 days [62]. Thus, the intake of β-cryptoxanthin was found to have preventive effects on STZ-diabetic state and bone loss in STZ-diabetic rats.

Bone loss is induced after ovriectomy (OVX), which is a model of postmenopausal osteoporosis. β-Cryptoxanthin (50 or 100 μg/kg body weight) was orally administered once daily for 3 months to OVX rats. The analysis using peripheral quantitative computed tomography shows that OVX induced a significant decrease in mineral content and mineral density in the femoral-diaphyseal and -metaphyseal tissues [63]. These decreases were prevented after the administration of β-cryptoxanthin (50 or 100 μg/kg). Moreover, OVX induced a decrease in bone biochemical components. These decreases are completely prevented after the administration of β-cryptoxanthin (50 or 100 μg/kg). β-Cryptoxanthin had a preventive effect on OVX-induced bone loss in vivo [63].

### Intake of dietary β-cryptoxanthin has a preventive effect in menopausal women

The effect of β-cryptoxanthin on bone metabolism in human is shown by using serum bone metabolic markers. Serum bone-specific alkaline phosphatase and γ-carboxylated osteocalcin are bone metabolic markers of osteoblastic bone formation [64,65]. Serum bone TRACP and *N*-telopeptides of type I collagen are metabolic markers of osteoclastic bone resorption [66,67]. The effect of prolonged intake of juice prepared from Satsuma mandarin (*Citrus unshiu *MARC) containing β-cryptoxanthin is shown by using circulating biochemical markers of bone metabolism in subjects including menopausal woman [68-70].

Twenty-one volunteers (10 males and 11 females) were divided into two groups of ten volunteers (5 males and 5 females) and eleven volunteers (5 males and 6 females). Each group was given sequentially juice (192 ml) containing two different contents of β-cryptoxanthin once a day for 28 or 56 days either a regular juice with naturally occurring 802 μg β-cryptoxanthin/100 ml or a reinforced juice containing 1500 μg β-cryptoxanthin/100 ml [68].

The intake of regular juice for 28 or 56 days in healthy subjects caused a significant increase in serum γ-carboxylated osteocalcin concentration, and the intake for 56 days produces a decrease in serum bone TRACP activity [68]. Moreover, the intake of the β-cyptoxanthin reinforced juice for 28 or 56 days caused an increase in serum γ-carboxylated osteocalcin concentration and a corresponding decrease in serum bone TRACP activity and *N*-telopeptide of type I collagen [68]. These findings suggest that the intake of β-cyptoxanthin reinforced juice has a stimulatory effect on osteoblastic bone formation and inhibitory effect on osteoclastic bone resorption in normal individuals [68].

The serum β-cyptoxanthin concentration was increased after the intake of regular juice for 56 days [69]. This increase was enhanced after the intake of β-cyptoxanthin-reinforced juice. The intake of regular juice or of β-cyptoxanthin-reinforced juice for 56 days caused an increase in serum γ-carboxylated osteocalcin and a decrease in serum bone TRACP activity [69]. A possible relationship between serum β-cyptoxanthin and circulating γ-carboxylated osteocalcin concentrations was found by using the value obtained from all groups for before intake and with the intake of regular juice and β-cyptoxanthin-reinforced juice. A negative relationship between serum β-cyptoxanthin concentation and circulating TRACP activity was observed [69]. This study shows that a relationship between serum β-cyptoxanthin and circulating bone metabolic markers is found in healthy individuals with the intake of juice containing β-cyptoxanthin.

Ninety volunteers, aged 27-65 years (19 men and 71 women), were enrolled in this study [70]. The seventy-one females included 35 premenopausal women (ages, 27-50 years) and 36 menopausal women (ages, 46-65 years). Volunteers were divided into four groups; placebo juice without β-cyptoxanthin (5 men and 19 women), juice containing β-cyptoxanthin at 1.5 mg/200 ml of juice/day (4 men and 17 women), 3.0 mg/day (5 men and 17 women), and 6.0 mg/day (5 men and 18 women). Placebo or juice (200 ml) was ingested once a day for 28 or 56 days.

Serum β-cryptoxanthin concentrations were increased after the intake of juice containing β-cryptoxanthin (1.5, 3.0, or 6.0 mg/day) for 28 or 56 days, and the increases were dose-dependent [65]. An increase in serum β-cryptoxanthin concentration was also observed at 28 days at the end of intake, indicating that the carotenoid is stable in the serum. Serum β-cryptoxanthin concentration was in the range of 4.20 × 10^-7 ^M to 4.89 × 10^-7 ^M in the placebo groups. The intake of juice reinforced with β-cryptoxanthin concentration at doses of 1.5, 3.0, or 6.0 mg/day increased the serum concentration to 2.43 × 10^-6^, 4.06 × 10^-6^, or 5.38 × 10^-6 ^M, respectively [70]. These increases were about 5 or 10 fold as compared with the value obtained before intake or after placebo intake. It has been shown that the serum concentration of β-cryptoxanthin increased due to the consumption of vegetable juice in women from 1.3 × 10^-7 ^to 5.3 × 10^-7 ^M [33].

In ninety volunteers (aged 27-65 years), serum bone-specific alkaline phosphatase activity was increased after the intake of juice containing β-cryptoxanthin (3.0 or 6.0 mg/day) for 56 days as compared with the value obtained before intake [70]. γ-Carboxylated osteocalcin concentration was increased after the intake of juice containing β-cryptoxanthin (3.0 or 6.0 mg/day) for 28 or 56 days as compared with the value obtained before intake or after the intake of placebo juice [70]. Serum TRACP activity and type I collagen *N*-telopeptide concentration were decreased after the intake of juice containing β-cryptoxanthin (3.0 or 6.0 mg/day) for 28 or 56 days as compared with the value obtained before intake or after intake of placebo juice, and significant decreases were also seen after the intake of 1.5 mg/day β-cryptoxanthin as compared with the value obtained before intake [70].

In menopausal women (36 volunteers), bone-specific alkaline phosphatase activity and γ-carboxylated osteocalcin concentration were increased after the intake of juice containing β-cryptoxanthin (3.0 or 6.0 mg/day) for 56 days as compared with the value obtained after placebo intake [70]. Also, this intake caused a decrease in bone TRACP activity and type I collagen *N*-telopeptide concentration. Thus, the prolonged intake of β-cryptoxanthin-reinforced juice has been demonstrated to have stimulatory effects on osteoblastic bone formation and inhibitory effects on osteoclastic bone resorption in menopausal women.

Meanwhile, serum calcium, inorganic phosphorous, and parathyroid hormone (intact) were not changed after the intake of β-cryptoxanthin-containing juice for 28 or 56 days. Other serum biochemical findings were not changed after the intake of juice containing β-cryptoxanthin (3.0 or 6.0 mg/day) for 56 days. The safety of β-cryptoxanthin in human is confirmed [70].

As mentioned above, the intake of juice reinforced with β-cryptoxanthin (3.0 or 6.0 mg/day) has been found to have an effect on circulating bone metabolic markers in men, premenopausal women, and menopausal women [70]. This indicates that the effects of β-cryptoxanthin in stimulating bone formation and inhibiting bone resorption are present in both sexes. Interestingly, the intake of juice reinforced with β-cryptoxanthin (3.0 or 6.0 mg/day) has been found to have effects on circulating bone metabolic markers in menopausal women, indicating that the supplementation of β-cryptoxanthin has preventive effects on bone loss due to osteoporosis in menopausal women. This preventive effect is obvious at a dose of β-cryptoxanthin of 3.0 mg/day in menopausal women. This dose may be suitable in the prevention of osteoporosis in human subjects.

Thus, the intake of reinforced juice, which contains more β-cryptoxanthin than regular juice, was demonstrated to have a preventive effect on bone loss that accompanies an increase in age.

### β-Cryptoxanthin and bone health: Epidemiological evidence

On the based on our findings, epidemiological studies support the view that the intakes of fruit and vegetables containing β-cryptoxanthin may reduce the risk of osteoporosis [71-73].

The effect of dietary antioxidants on knee structure in a cohort of healthy, middle-aged subjects with no clinical knee osteoarthritis is reported [71]. Two hundred and ninety-three healthy adults (mean age = 58.0 years) without knee pain or knee injury were selected from an existing community-based cohort. The intake of antioxidant vitamins and food sources by these individuals was estimated from a food frequency questionnaire at baseline. The cartilage volume, bone area, cartilage defects and bone marrow lesions were assessed approximately 10 years later using magnetic resonance imaging. Higher vitamin C intake was associated with a reduced risk of bone marrow lesions and with a reduction in the tibial plateau bone area. There was an inverse association between fruit intake and the tibial plateau bone area and between fruit intake and the risk of bone marrow lesions. Neither fruit intake nor vitamin C intake was significantly associated with the cartilage volume or cartilage defects.

Lutein and zeaxanthin intake was associated with a decreased risk of cartilage defects, and vitamin E intake tended to be positively associated with the tibial plateau bone were only after adjusting for vitamin C intake. The β-cryptoxanthin intake was inversely associated with the tibial plateau bone area after adjusting for vitamin E intake. These observations suggest a beneficial effect of fruit consumption and vitamin C intake as they are associated with a reduction in bone size and the number of bone marrow lesions, both of which are important in the pathogenesis of knee osteoarthritis [71].

Bone mineral density (BMD) in post-menopausal female subjects has been shown to associate with serum antioxidant carotenoids. A total of six hundred ninety-nine subjects (222 males and 477 females) who had received health examinations in the town of Mikkabi, Shizuoka Prefecture, Japan, participated in the study [72]. Radial BMD was measured by using dual-energy X-ray absorptiometry. The associations of serum carotenoid concentrations with the dadial BMD were evaluated cross-sectionally. In male and pre-menopausal female subjects, the six serum carotenoids were not associated with the radial BMD. On the other hand, in post-menopausal female subjects, serum β-cryptoxanthin and β-carotene were weakly but positively correlated with the radial BMD. After adjustment for confounders, the odds ratio (OR) for the lowest quartile of BMD in the high groups of serum β-cryptoxanthin against the lowest quartile was 0.45 in post-menopausal female subjects. However, this association was not significant after further adjusting for intakes of minerals and vitamins. Antioxidant carotenoids, especially β-cryptoxanthin, significantly but partly associated with the radial BMD in post-menopausal female subjects [72].

Seasonal variation of serum α- and β-cryptoxanthin and 25-OH-vitamin D_3 _in women with osteoporosis is showed [73]. In six hundred fourty-four women with osteoporosis, serum β-cryptoxanthin and 25-OH-vitamin D_3 _showed a weak but significant correlation and exhibited a complementary seasonal distribution [73]. Dietary intake and serum levels of β-cryptoxanthin have been inversely related to different bone and joint disorders and in vitro and animal studies have shown that β-cryptoxanthin displays a unique anabolic effect on bone calcification. Due to the emerging role of β-cryptoxanthin in bone biology, this study was aimed to assess the serum distribution and variability of β-cryptoxanthin and their potential relation to 25-OH-vitamin D_3 _in women with osteoporosis [73].

Overall, significant seasonal variations were found for the three analyses and inter-individual variation was also high (60-73%). β-Cryptoxanthin and 25-OH- vitamin D_3 _exhibited a marked complementary seasonal distribution in serum, with vitamin D displaying the highest values in summer and β-cryptoxanthin in winter.

Given the anabolic effect of β-cryptoxanthin on bone calcification and its complementary seasonal distribution with respect to 25-OH- vitamin D_3_, the potential role of β-cryptoxanthin as a sustainable nutritional approach to improving bone health deserves to be further evaluated [73].

### Dietary total carotenoids and osteoporosis prevention

Carotenoid β-cryptoxanthin was demonstrated to stimulate bone formation and to suppresse bone resorption in vitro and in vivo studies. Effect of the associations of total and individual carotenoid intake (α-carotene, β-carotene, β-cryptoxanthin, lycopene, lutein, and zeaxanthin) with incident hip fracture and nonvertebral osteoporotic fracture are examined [74]. Three hundred seventy men and 576 women (mean age, 75 ± 5 years) from the Framingham Osteoporosis Study completed a food frequency questionnaire (FFQ) in 1988-1989 and were followed for hip fracture until 2005 and nonvertebral fracture until 2003. Tertiles of carotenoid intake were created from estimates obtained using the Willett FFQ adjusting for total energy (residual method). HRs were estimated using Cox-proportional hazards regression, adjusting for sex, age, body mass index, height, total energy, calcium and vitamin D intake, physical activity, alcohol, smoking, multivitamin use, and current estrogen use. A total of 100 hip fractures occurred over 17 years of follow-up. Subjects in the highest textile of total carotenoid intake had lower risk of hip fracture. Subjects with higher lycopene intake had lower risk of hip fracture and nonvertebral fracture. A weak protective trend was observed for total β-carotene for hip fracture alone, but associations do not reach statistical significance. No significant associations are observed with α-carotene, β-cryptoxanthin, or lutein plus zeaxanthin. These findings suggest a protective role of several carotenoids for bone health in older adults [74].

Antioxidant defenses may be compromised in osteoporotic women. Little is known about fruit and vegetable or carotenoid consumption among postmenopausal women. The primary carotenoids in human serum are α- and β-carotene, lycopene, β-cryptoxanthin, lutein and zeaxanthin [75]. The interrelationships among serum carotenoid concentrations, fruit and vegetable intake, and osteoporosis in postmenopausal women (n = 59, 62.7 +/- 8.8 years) are shown [75]. Bone density was assessed by dual energy x-ray absorptiometry (DEXA) and osteoporosis diagnosis was based upon T-scores. Serum samples (n = 53) and three-day diet records (n = 49) were analyzed. Logistic regression analyzed differences between carotenoids after adjusting for serum retinol; supplement usage; milk, yogurt, fruit, and vegetable intake; and body mass index. Pearson statistics correlated carotenoids with specific fruit or vegetable intake. Serum lycopene concentrations were lower in the osteoporosis group than controls. β-Cryptoxanthin intake was higher in the osteoporosis group. Total fruit and vegetable intakes were correlated with serum lycopene and β-cryptoxanthin [75].

Recent studies show that antioxidants may reduce the risk of osteoporosis. The associations of BMD with dietary patterns of antioxidant vitamins and carotenoids are shown [76]. A total of 293 post-menopausal female subjects who had received health examinations in the town of Mikkabi, Shizuoka Prefecture, Japan, is participated in the study [76]. Radial BMD was measured using DEXA. Dietary patterns were identified on a selected set of antioxidants through principal component factor analysis. Three dietary patterns are identified. The "retinol" pattern, characterized by notably high intakes of preformed retinol, zeaxanthin, and vitamin E, is positively associated with the risk for low BMD [76].

In contrast, the " β-cryptoxanthin" pattern, characterized by notably high intakes of β-cryptoxanthin and vitamin C, is negatively associated with low BMD [76]. The odds ratios for low BMD in the highest tertiles of dietary intakes of preformed retinol, vitamin C, and β-cryptoxanthin against the lowest tertiles are 3.22 [95% confidence interval (CI), 1.38-7.51], 0.25 (CI, 0.10-0.66), and 0.40 (CI, 0.17-0.92), respectively, after adjustments for confounders [76]. However, negative associations of vitamin C and β-cryptoxanthin with low BMD were not significant after further adjustment for intake of β-cryptoxanthin or vitamin C, respectively [76]. Higher intakes of both vitamin C and β-cryptoxanthin were significantly associated with low BMD [76]. The findings suggest the combination of vitamin C and β-cryptoxanthin intakes might provide benefit to bone health in post-menopausal Japanese female subjects. The combination of vitamin C and β-cryptoxanthin may be associated with radial BMD in post-menopausal women.

It is possible that the preventive effect of β-cryptoxanthin on osteoporosis is enhanced with other carotenoids and factors in foods. This may be important in maintaining bone health in human life with food intake.

## Conclusion

Bone mass is changed with increasing ages. Bone loss is dramatically induced in postmenopausal women. Nutritional and food factors may have a preventive role in the decrease in bone mass with aging and pathophysiologic conditions. Among various carotenoids, β-cryptoxanthin has been found to have a unique anabolic effect on bone mass due to stimulating osteoblastic bone formation and inhibiting osteoclastic bone resorption. β-Cryptoxanthin modulates gene expression of various proteins that involve in osteoblastic bone formation and osteoclastic bone resorption. β-Cryptoxanthin may bind to orpharn receptors, showing a novel mechanism of the carotenoid in the aspect of bone fields. Further mechanism of β-cryptoxanthin remains to be elucidated, however.

The intake of dietary β-cryptoxanthin has been shown to have preventive effect on bone loss in animal models for osteoporosis and in menopausal women, suggesting the possibility of pharmacological use of β-cryptoxanthin in prevention and therapy of osteoporosis and other bone diseases. The supplemental intake of β-cryptoxanthin with higher dose may have a pharmacologic role in the therapy of osteoporosis with clinical studies. In addition, potential effects with β-cryptoxanthin derivatives are expected in the development of new drug for treatment of bone diseases.

In addition, the role of β-cryptoxanthin in bone health has been also shown in human subjects with epidemiological studies. The supplemental intake with the combination of β-cryptoxanthin and other nutritional factors may has a potential effect in the maintaining of bone health and decrease in bone loss.

## Competing interests

The author declares that they have no competing interests.

## Authors' contributions

MY rafted the manuscript and read and approved the final manuscript.
